# Functional Green-Tuned Proteorhodopsin from Modern Stromatolites

**DOI:** 10.1371/journal.pone.0154962

**Published:** 2016-05-17

**Authors:** Virginia Helena Albarracín, Ivana Kraiselburd, Christian Bamann, Phillip G. Wood, Ernst Bamberg, María Eugenia Farias, Wolfgang Gärtner

**Affiliations:** 1 Planta Piloto de Procesos Industriales y Microbiológicos (PROIMI), CCT, CONICET. Av. Belgrano y Pasaje Caseros. 4000- S. M. de Tucumán, Argentina; 2 Facultad de Ciencias Naturales e Instituto Miguel Lillo, Universidad Nacional de Tucumán, 4000, S. M. de Tucumán, Argentina; 3 Instituto de Biología Molecular y Celular de Rosario (IBR - CONICET), Facultad de Ciencias Bioquímicas y Farmacéuticas (FBIOYF - UNR), Suipacha 590, 2000, Rosario, Santa Fe, Argentina; 4 Max-Planck-Institute for Biophysics, Max-von-Laue-Straße 3, D-60438 Frankfurt am Main, Germany; 5 Max-Planck-Institute for Chemical Energy Conversion, Stiftstrasse 34–36, D-45470 Mülheim, Germany; Friedrich Schiller University, GERMANY

## Abstract

The sequenced genome of the poly-extremophile *Exiguobacterium* sp. S17, isolated from modern stromatolites at Laguna Socompa (3,570 m), a High-Altitude Andean Lake (HAAL) in Argentinean Puna revealed a putative proteorhodopsin-encoding gene. The HAAL area is exposed to the highest UV irradiation on Earth, making the microbial community living in the stromatolites test cases for survival strategies under extreme conditions. The heterologous expressed protein E17R from *Exiguobacterium* (248 amino acids, 85% sequence identity to its ortholog ESR from *E*. *sibiricum*) was assembled with retinal displaying an absorbance maximum at 524 nm, which makes it a member of the green-absorbing PR-subfamily. Titration down to low pH values (eventually causing partial protein denaturation) indicated a pK value between two and three. Global fitting of data from laser flash-induced absorption changes gave evidence for an early red-shifted intermediate (its formation being below the experimental resolution) that decayed (τ_1_ = 3.5 μs) into another red-shifted intermediate. This species decayed in a two-step process (τ_2_ = 84 μs, τ_3_ = 11 ms), to which the initial state of E17-PR was reformed with a kinetics of 2 ms. Proton transport capability of the HAAL protein was determined by BLM measurements. Additional blue light irradiation reduced the proton current, clearly identifying a blue light absorbing, M-like intermediate. The apparent absence of this intermediate is explained by closely matching formation and decay kinetics.

## Introduction

Microbial rhodopsins are considered to be the simplest energy-harvesting photoreceptors consisting of a single, membrane-embedded protein covalently binding a retinal (Vitamin-A aldehyde) chromophore [1]. The study of the microbial archetype, bacteriorhodopsin (BR) from the archaeon *Halobacterium salinarum* [2], led to the suggestion that BR evolved as a consequence of exposure to the extreme halophilic, oxygen-deprived environment of *H*. *salinarum*. However, further surveys demonstrated the worldwide existence of many microbial rhodopsins with a spectrum of functions in ecological niches, thus being probably the most abundant phototrophic system on Earth [3].

Out of this versatile family of proteins, the proteorhodopsins (PRs), bacterial ion pumps, represent the largest group [3]. They were mostly found in microbial communities living in environments where solar exposure is high i.e. the upper layer of oceans, freshwater, brackish and hypersaline aquatic ecosystems [4–18], sea-ice matrix [19], non-marine permafrost [20, 21] and phyllosphere [22]. Moreover, proteorhodopsin genes are widely distributed among divergent bacterial taxa included alpha, beta, delta and gamma proteobacteria, Bacteroidetes, Flavobacteria, Firmicutes, Deinococcus-Thermus, Chloroflexi, Planctomycetes and Actinobacteria [23–28].

Detailed molecular studies on various PRs and their three-dimensional structure [21, 29] (in addition to the enormous information on structure and function of BR) have generated a precise picture of their light-induced reactions [3]. Key amino acids, already identified as instrumental for the function of BR have been allocated also to the PRs [30–32]. A further evolutionary aspect of PRs was detected from sequence alignments, together with spectroscopic measurements. Two subfamilies of PRs were identified, distinguished by their absorbance maxima [33–35]. The group coined GPRs (green-absorbing) showed absorbance maxima around 525 nm, whereas the other group near 490 nm (BPRs, blue-absorbing). This switch in absorbance maxima is accomplished by a single amino acid exchange and is understood as an adaptation to the prevailing spectral conditions at different depths in a water column with green light dominating close to the surface and solely blue light remaining at greater water depths [33–35].

Microbes might profit from PRs in a variety of ways; planktonic ones may gain a competitive advantage by using these photoproteins to generate a light-driven proton gradient for the synthesis of ATP [36, 37]. In extreme environments though, PRs may even be paramount to counteract starvation and other hostile conditions thanks to the potential for harvesting extra energy by means of phototrophy [16, 19].

A characteristic example of a highly irradiated, extreme environment is the High-Altitude Andean Lakes (HAAL) area in the Central Andes region in South America [38, 39]. These shallow lakes and salterns dispersed at altitudes above 3,000 m are exposed to a unique combination of severe conditions including high solar global and UV irradiation, hypersalinity, wide fluctuations in daily temperatures, desiccation, high pH, and high concentrations of toxic elements including arsenic, which have brought forth a distinguished microbiodiversity of extremophiles [38–42,62,63].

Among microbialites of the HAAL [41, 43–47], modern stromatolites were first described as thriving in the shore of Laguna Socompa, a hypersaline and moderate alkaline lake located at 3,570 m at the base of the active Socompa volcano. These stromatolites (41) exhibit an atypical microbial community with abundant representatives of Deinococcus-Thermus, Rhodobacteraceae, Desulfobacterales and Spirochaetes. Their metagenome revealed a high proportion of sequences that could not be classified at phylum level, depicting less than 80% identity to the best hit in the NCBI database, suggesting the presence of novel distant lineages [41]. Thus, the study of microbes associated with Socompa stromatolites and their environment could provide further understanding of their adaptation and the function of proteins working under extremely harsh conditions.

Several bacterial strains from Socompa stromatolites were isolated, physiologically characterized and subjected to genome pyrosequencing [18, 48, 49]. Screening of the genomes of these strains i.e. *Exiguobacterium* sp. S17, *Sphingomonas* sp. S17 and *Salinivibrio* spp. S10B and S34, yielded genes putatively encoding essential traits for survival under multiple environmental extreme conditions, e.g., high levels of UV radiation, elevated salinity, and the presence of poisoning arsenic concentrations [18, 45, 48–50]. Likewise, genes putatively coding for the bacteriorhodopsin protein family were found showing sequential features indicating the presence of proteorhodopsins and xantorhodopsins [18, 45–47]. The former findings called for a more detailed, molecular investigation of the mechanisms involved in the resistance of these strains to extreme but common impairing factors in its original environment. We herein present the first functional characterization of E17R, a PR from *Exiguobacterium* sp. S17, a halotolerant, highly arsenic resistant extremophile isolated from a modern stromatolite, located at an altitude of more than 3,500 m above sea level.

## Materials and Methods

### Strain, media and culture conditions

**Fig 1:**
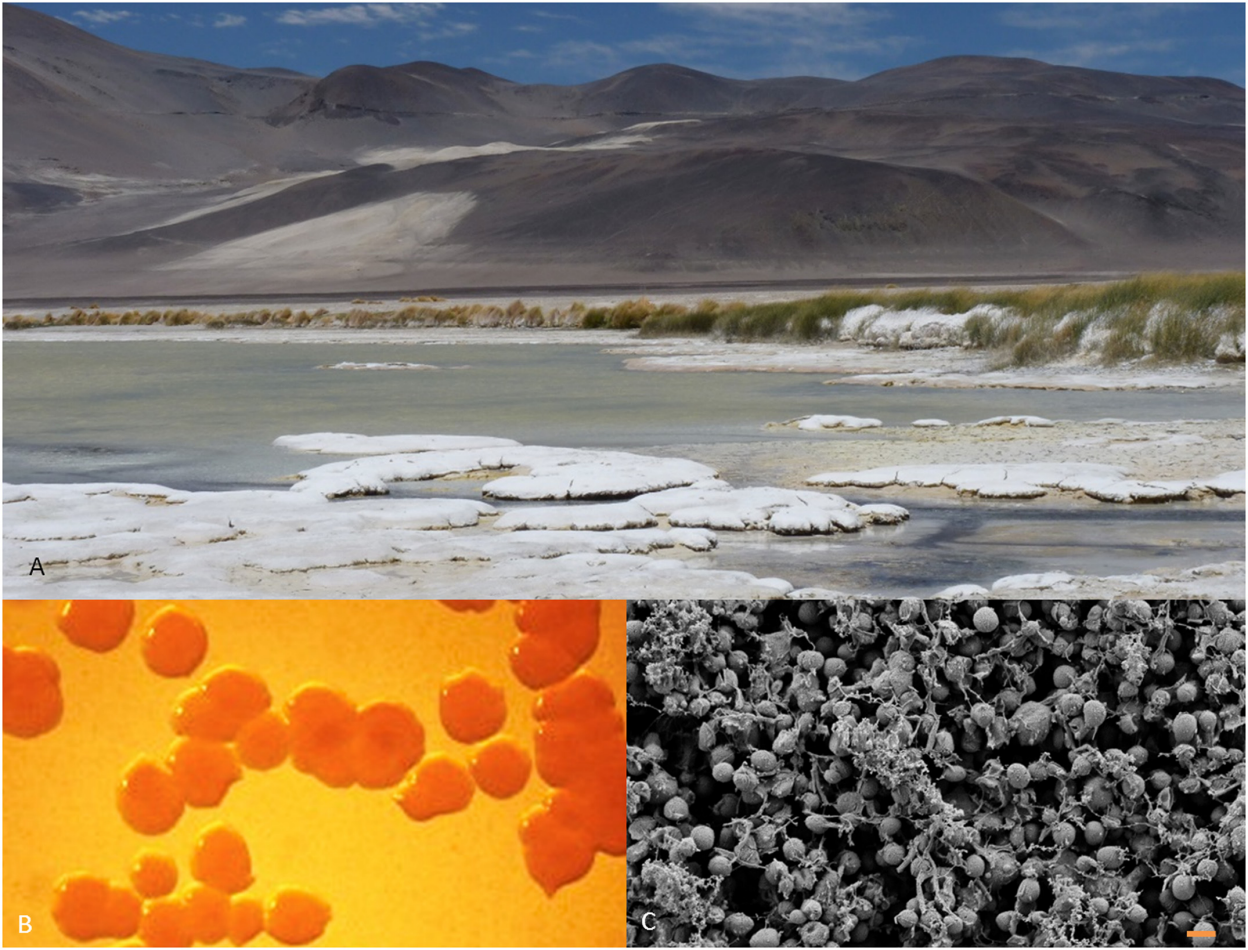
A. Socompa Stromatolites at Laguna Socompa, the original habitat of *Exiguobacterium* sp. S17. B. Macroscopic appearance of S17 colonies. C. Scanning electron micrograph of S17 cells. Bar: 1 μm.

The extremophile *Exiguobacterium* sp. S17 was previously isolated from modern stromatolites located at the shore of Lake Socompa (3,570 m; Fig 1) [50], and is currently maintained in the culture collection of Laboratory of Microbial Research on Andean Lakes (National System of Biological Data). For preculture and DNA extraction, S17 was grown in LB media or on LB-agar plates (15.0 g/L).

### DNA isolation

Genomic DNA from *Exiguobacterium* sp. S17 was purified from cells grown on LB broth for 24 h at 30°C and harvested by centrifugation (3,000 *g* for 10 min at 4°C). The pellets were washed twice with distilled water. Total genomic DNA was extracted with the DNeasy Blood and Tissue Kit (Qiagen) using the specific protocol for Gram-positive bacteria following the manufacture’s recommendations.

### Gene cloning

*E*. *coli* strain XL-1 Blue (Stratagene) was used for all DNA manipulations. To amplify the putative PR gene from *Exiguobacterium* sp. S17, two gene-specific primers PR-S17F 5′ GATCGAATTCG**ATG**GACGAAGAAGTCAATTTAC and PR-S17R 5′ GATTCTCGAGCGTTTTAATTTGTTTGAGCGTCGCGACGCTCAAACAAATTAAAACG were designed according to the published genomic DNA sequence of S17 [48]. The gene start codon (ATG) is shown in bold-type in the forward primer. Restriction sites for EcoRI and XhoI were introduced in the forward and reverse primers (underlined). E17R -gene was PCR amplified and cloned into the pET-26b(+) vector (Novagene). The vector was transformed into *Escherichia coli* XL1 blue cells for further analysis.

### Phylogenetic analysis of protein sequences

The sequence of the E17R protein from *Exiguobacterium* sp. S17 is available in GenBank (WP_016509804) together with the draft genome obtained by pyrosequencing (ASXD01). All sequences of PR-related proteins with significant homology (S1 File) were retrieved from public databases via the National Center for Biotechnology Information web site (www.ncbi.nlm.nih.gov). Multiple sequence alignments and phylogenetic analyses were carried out using MUSCLE [51] and Phylogeny.fr [52, 53]. The evolutionary history was inferred using the Maximum likelihood method [54]. The tree was drawn to scale, with branch lengths in the same units as those of the evolutionary distances used to infer the phylogenetic tree. The percentages of replicate trees in which the associated taxa cluster together in the bootstrap test (1,000 replicates) are shown next to the branches. The analysis involved 22 amino acid sequences of PR-genes of *Exiguobacterium* strains. All positions containing gaps and missing data were eliminated.

### Genomic environment analysis of proteorhodopsin

Genes for microbial rhodopsin and its genomic environment were identified using the RAST annotation platform [55] and the IMG analysis system [56]. The search and comparison was performed with all genomes available for the *Exiguobacterium* genus.

### Protein expression and purification

Heterologous expression was performed with *E*. *coli* BL21 (DE3) cells transformed with the recombinant vector pET26-E17R. Cells from an overnight culture were used to inoculate a 200 mL culture, which then was used to inoculate 6 L of LB medium. Cells were grown with 40 μg/mL kanamycin, until an OD_600_ of 0.6 was reached. At that point, expression was induced with 0.5 mM IPTG. Cells were harvested by centrifugation after 12 h of incubation at 30°C. Cells were resuspended in 50 mM Tris, 5 mM MgCl_2_ pH 8.5 added with 1 mM PMSF, 5 μM retinal (final concentrations) and DNase. Cells were then stirred for 1 h at 4°C and then lysed using a microfluidizer (Microfluidics Corporation, M110LA). The cell lysate was centrifuged for 1 h at 186,000 *g* at 4°C. The pellet was resuspended in 20 mM HEPES, 100 mM NaCl, pH 7.4, added with 5 μM retinal and 1% (w/v) n-dodecyl-β-maltoside (DDM) (final concentrations). Following a second centrifugation (1 h at 186,000 *g*, 4°C), the supernatant was filtered and proteorhodopsin was obtained by IMAC purification using a His-tag (ÄKTA, GE Healthcare).

### Steady state absorbance and fluorescence spectroscopy

Steady state spectra were recorded at room temperature either on a Hitachi U3000 spectrophotometer or on a Hitachi F-4500 spectrofluorometer. The extinction coefficient of E17R was determined from the difference spectrum of a sample before and after treatment with 10 mM hydroxylamine (final concentration) and illumination (>480 nm) at room temperature. The value for E17R can be quantified from the known extinction coefficient of the retinal oxime, ε_max_ = 33’600 M^-1^ cm^-1^ [57].

### pH-titration

For pH-tritation experiments, proteorhodopsin samples were adjusted to *A*_540_ = 0.1–0.5 in a buffer containing 20 mM HEPES, 100 mM NaCl, and 0.1% (w/v) DDM at pH 7.4. The titration was carried out in a 1 × 1 cm cuvette with continuous stirring. The pH values were adjusted with 1 M NaOH or 1 M HCl, before the spectra were recorded (Cary50, Varian, USA). Reversibility was checked at the end of the titration. Data were fitted using the Henderson-Hasselbalch equation.

### Flash photolysis

Samples were prepared with identical buffer conditions as for the pH titration with pH = 7.4. Transient absorbance changes were recorded on a home-built flash-photolysis setup. The reaction was started by a 10 ns laser pulse from an excimer laser pumped dye laser (Coumarin 307, λ = 503 nm, 5–10 mJ cm^-2^). Light from 75 W XBO lamp was filtered by narrow-band interference filters and passed through the sample and a monochromator, before it was detected by a photodiode. Absorbance changes were followed over time on two oscilloscopes for the following wavelengths: 380, 400, 420, 440, 460, 480, 500, 517, 540, 562, 580, 600, 620, 645 nm. The number of data points was reduced by a logarithmic interpolation procedure before the data was analyzed by a global fitting routine. To this end, data blocks of centuries are averaged over 2^(n-1)^ points per n^th^ century yielding 880 data points for the initial 45k points of the raw data for each of the oscilloscope with sampling rates of 200 ns per point and 20 μs per point, respectively. The two datasets were combined yielding 1520 point covering a range up to 1 second.

### Electrophysiology of reconstituted E17R in liposomes

Proteorhodopsin was reconstituted into liposomes. Liposomes were formed from *E*. *coli* polar lipids, resuspended in 20 mM HEPES, 100 mM NaCl, and 2% (w/v) n-octyl-β-glucopyranoside (OG) at pH 7.4, to which 7.5 μM protein was added. The resulting mixture was incubated on ice for 15 min. Detergent-adsorbing beads (BioBeads, BioRad, Germany) were added to the solubilized protein and the solution was stirred overnight at 4°C.

Black Lipid Membranes (BMLs) were formed across an orifice (area ~0.5×10^−3^ cm^2^) between two compartments of a Teflon cuvette filled with a buffer containing 50 mM Tris, 5 mM MgCl_2_, pH 8.5. The membrane-forming solutions contained 1.5% (w/v) diphytanoyl-phosphatidylcholine (Avanti Biochemicals, Birmingham, AL) and 0.025% (w/v) octadecylamine (Riedel-de-Haën, Hannover, Germany) in n-decane to obtain a positively charged membrane. Proteorhodopsin-containing proteoliposomes were added to the compartment under gentle stirring. A combination of ionophores (2 μM monensin and FCCP) was added, which effectively permeabilizes the compound membrane system (final conductance 50–100 nS). A 100 W Osram HBO mercury arc lamp with a combination of bandpass filters removing UV and IR irradiation (<380 nm and >750 nm) and a cut-off filter removing irradiation <495 nm was used for sample illumination. Blue light effect was generated by additional irradiation with light longer than 360 nm. The measurements were carried out at room temperature. The photocurrents were recorded with pClamp9.0 software via a MiniDigi 1A digitizer (Axon Instruments).

## Results

### A green proteorhodopsin from *Exiguobacterium* sp. S17

**Fig 2:**
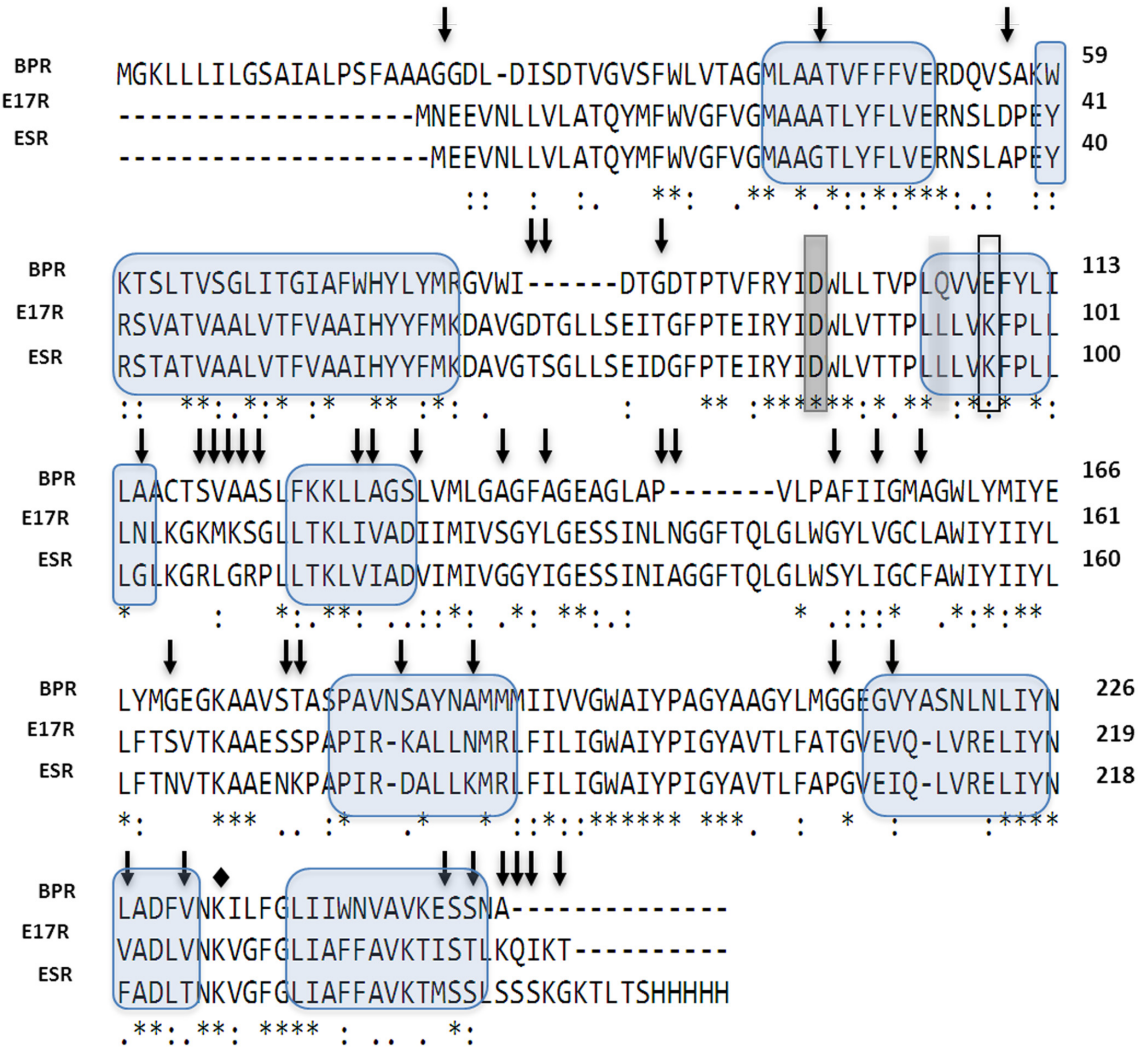
Multiple protein alignment of PR from *Exiguobacterium* sp. S17 (E17R), green-light absorbing proteorhodopsin from *Exiguobacterium sibiricum* (ESR) and blue-light absorbing proteorhodopsin from the uncultured gamma-proteobacterium “Hot 75m4” (BPR). Residues shared between all the protein variants are marked with asterisks. Single amino acid residue at position 106 (BPR numbering) that functions as a spectral tuning switch and accounts for most of the spectral difference between the two pigment families is highlighted in light grey. Primary proton acceptor and donor are highlighted in dark grey (D86) and with a frame (K97), respectively. The Schiff base (K232 for ESR, K226 for E17R) is indicated by a diamond. Residues differing between both green-PRs are indicated with arrows. The seven transmembrane α-helices are indicated with blue bubbles.

**Fig 3:**
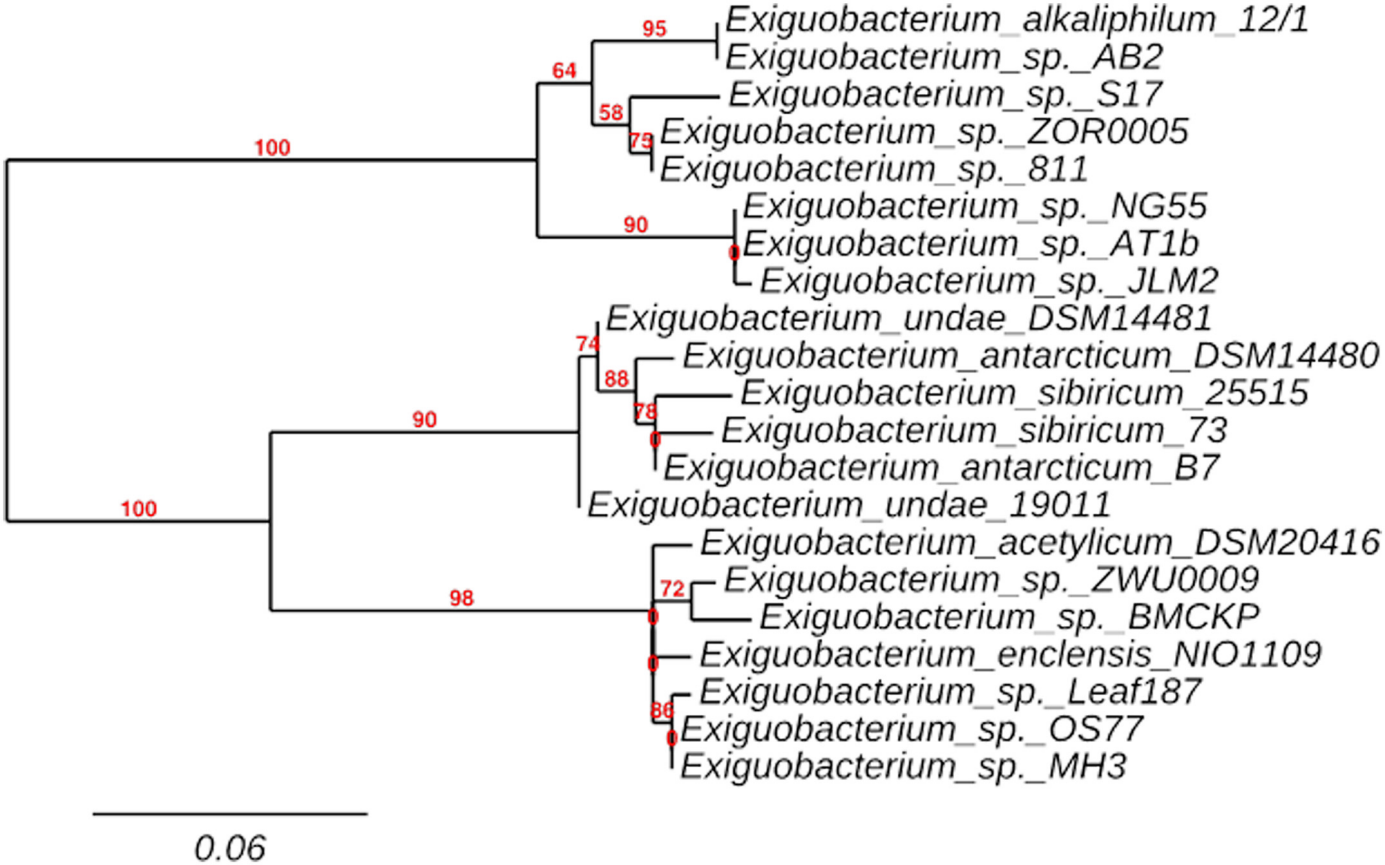
Phylogenetic tree inferred using the Maximum likelihood method of 21 amino acid sequences of *Exiguobacterium* spp. closely related with E17R. The percentage of replicate trees in which the associated taxa clustered together in the bootstrap test (1,000 replicates) are shown next to the branches. The tree is drawn to scale, with branch lengths in the same units as those of the evolutionary distances used to infer the phylogenetic tree.

**Fig 4:**
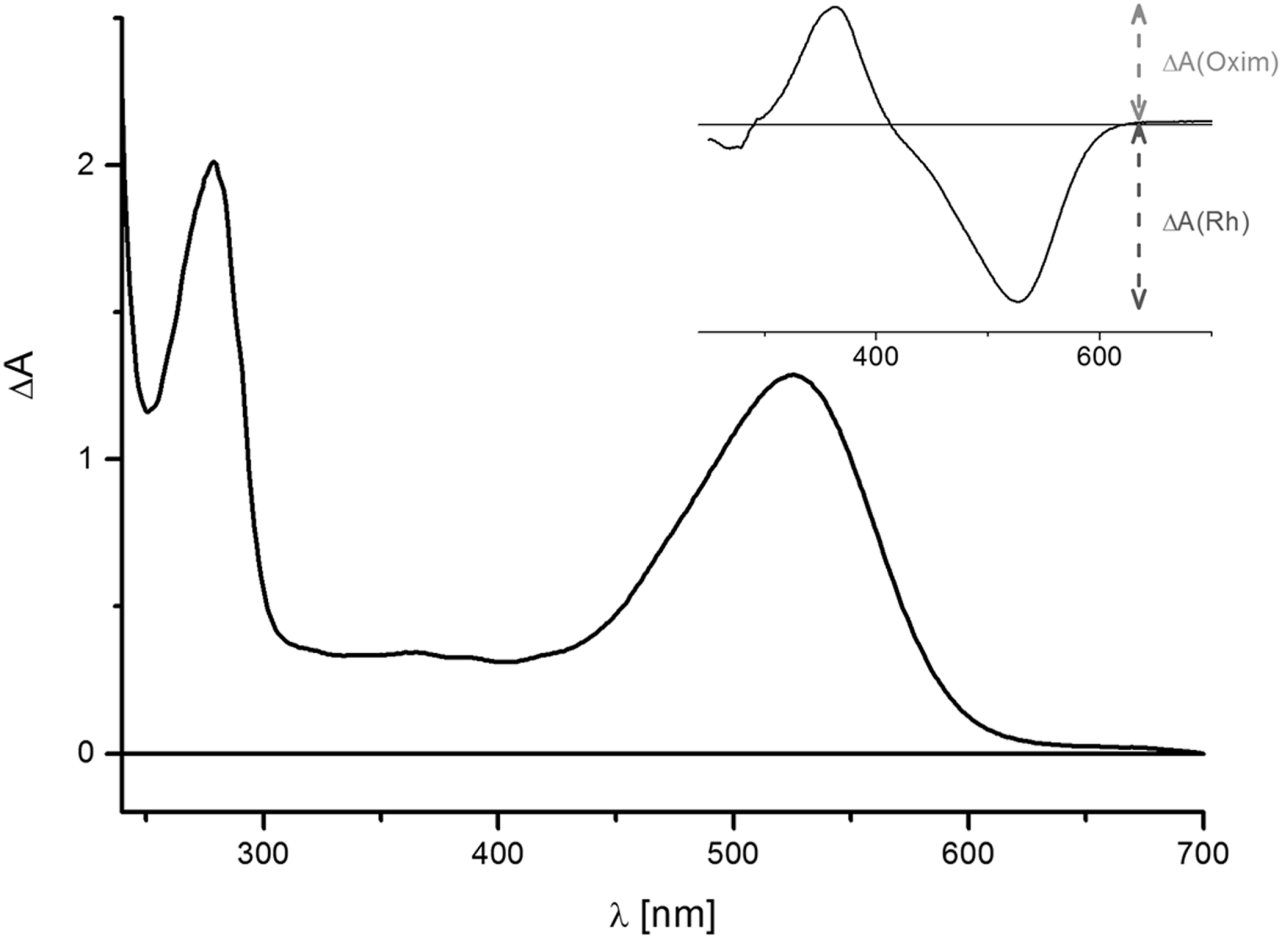
Absorption spectrum of E17R in 20 mM Hepes, 100 mM NaCl, 0.03% DDM. The inset shows the difference spectrum after bleaching with hydroxylamine. The extinction coefficient was calculated using the absorbance of the oxime product ΔA(oxime) in comparison to the bleached rhodopsin absorbance ΔA(Rh) as reference.

E17R, a gene predicted as belonging to the bacteriorhodopsin family (L479_RS10205/WP_016509804), was found in the genome of *Exiguobacterium* sp. S17. It encodes a protein of 248 amino acid residues (744 bp), E17R, with features typical of a microbial rhodopsin. All essential amino acid residues of the energy transducing rhodopsins are conserved (Figs 2 and 3). E17R has 85% sequence identity with the green-PR of the psycrophilic *Exiguobacterium sibiricum* (ESR) for which the crystal structure was recently solved (PDB: 4HYJ) [21]. Alignment of these two proteins sequences with that of the blue-light absorbing proteorhodopsin (BPR) of uncultured Gamma-proteobacterium Hot 75m4 (http://www.uniprot.org/taxonomy/245185) clearly showed the instrumental difference in an amino acid position associated with spectral tuning. Both *Exiguobacterium* proteins contain a leucine residue at position 94 (E17R, position 93 at ESR), which fine-tunes the PR absorption peak towards green light (absorption maximum 524 nm, Fig 4). This is in accordance with the shallow nature of the stromatolite formation in the shore of L. Socompa, the original environment of S17 (Fig 1). In turn, the corresponding position in BPR (106) is occupied by a glutamine, which is known to yield an absorption maximum near 490 nm [5, 32], a feature for proteorhodopsin-bearing microbes from deep-sea in coincidence with the light quality available at depths greater than 50 m.

A BLASTP search in all publicly available genomes of the *Exiguobacterium* genus was performed. Twenty-one genomes displayed a sequence with significant similarity (higher than 80%) to that of S17 proteorhodopsin (S1 File). E17R shares the highest sequence identity with *Exiguobacterium* sp. 8-11-1 (98%), *Exiguobacterium* sp. AB2 (95%) and *Exiguobacterium* sp. AT1b (94%). All PR sequences display Leu in position 106 (following the numbering of the BPR *Gamma-proteobacterium Hot 75m4*), assigning them to the “green-tuned” PR subfamily. A phylogenetic tree (Fig 3) constructed with the 22 related rhodopsins (S1 File) revealed two divergent clades, one including E17R (mostly form by alkaliphiles/thermophiles) and the other one mostly formed by psycrophiles/psycrotolerants including two branches, the one of *Exiguobacterium acetylicum* and the one of *E*. *sibiricum* ESR. Thus, in view of the phylogenetic distance from ESR, further biochemical and spectroscopic studies of the novel proteorhodopsin E17R were performed.

### Biochemical and steady-state spectral properties of E17R

Gene E17R (L479_RS10205/WP_016509804) encodes for a protein with 248 amino acids (MW: 27.4 kDa) that showed typical features of a microbial rhodopsin. Secondary structure prediction identified seven transmembrane α-helices, a lysine residue serving as putative retinal binding site at the seventh helix and proton donor and acceptor residues that identify this protein as a light-driven proton pump (Fig 2). Expression and purification of E17R yielded after purification and retinal addition a protein with λ_max_ = 524 nm (solubilized in 0.03% [w/v] DDM, Fig 4). The chromophore extinction coefficient was determined as 50,000 M^-1^ cm^-1^ from the difference spectrum after bleaching in the presence of hydroxylamine, which formed the retinal oxime (inset in Fig 4) [57]. The ratio of protein (280 nm) to chromophore (524 nm) absorbance can be taken as a purity factor and it amounts to ~1.3 (taking into account increased scattering around 280 nm). The value is slightly higher than the theoretical limit of ~1 as calculated from the extinction coefficients for the protein moiety and the chromophore (Fig 4).

**Fig 5:**
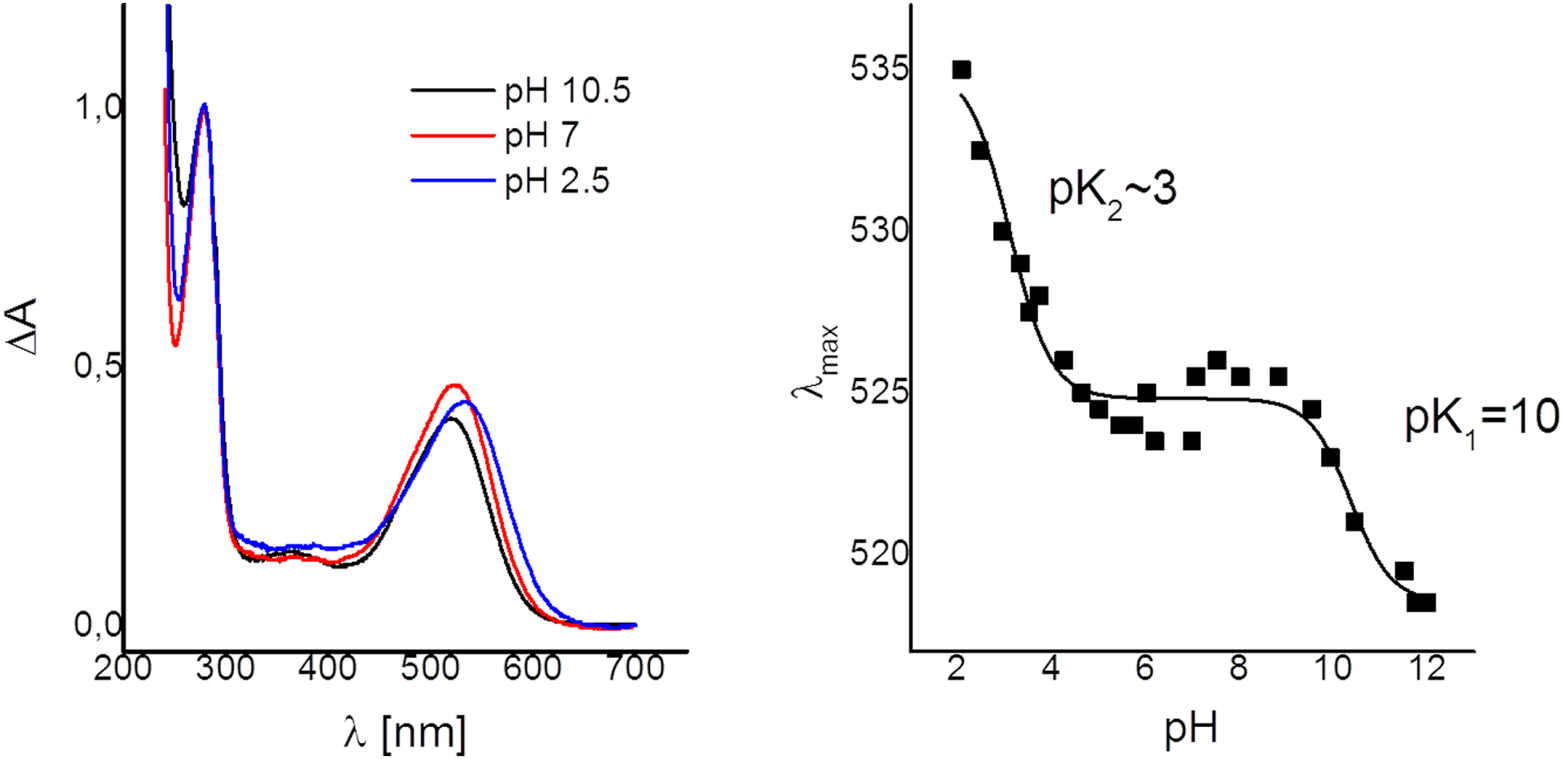
**A.** Absorbance spectra of E17R at three selected pH values. **B.** Titration curve as determined from the absorbance maxima.

We followed pH-induced absorbance changes in E17R between pH 2 and pH 10 in order to identify de- or reprotation of amino acids in close proximity to the chromophore (Fig 5). As reported for the closely related ESR [58], pH-titration reveals an ongoing red-shift of the absorption maximum upon acidification. Usually, this colour change is attributed to the protonation of the counterion of the protonated Schiff-base (D85 in ESR). pH-Titration experiments with E17R showed a similar absorbance change suggesting that D86 is the Schiff base counterion in E17R. pH-values lower than 2.0 could not be reached, as the protein lost stability irreversibly. In the accessible pH range, we observed a complex titration behaviour, similar to that described for ESR. For this protein, the data was interpreted as a strong interaction between the counter-ion D85 (D86 for E17R) and a conserved histidine residue (H57 in ESR, H58 in E17R) located in the second transmembrane helix (TMH B) [55]. For E17R, the fit of the absorbance changes shifting from 525 nm to 535 nm or higher gives a pK_2_~3 (Fig 5). Note that this is an approximate value, as the final state at acidic pH (<2) was not reached. The second transition is observed with a pK_1_ ~10 accompanied by smaller spectral changes (from 517 nm to 525 nm). The fluorescence data is in line with the argument given in Balashov et al. (2012) [58] for the assignment of the titratable groups. The major increase in fluorescence intensity observed at wavelengths longer than 620 nm can be attributed to the protonation of the proton acceptor D86 and takes place only at pH values <3 (data not shown). On the other hand, there is almost no change in fluorescence intensity at pH values >8. Therefore, the second transition might be assigned to deprotonation of the His interacting strongly with D86. It was proposed that this interaction keeps the counter-ion in its deprotonated state even at low pH values. Such behaviour is different from, e.g., the very first identified green-absorbing PR that has a pK_a_ ~7.5 for its counter-ion D100 [58].

### Flash photolysis

**Fig 6:**
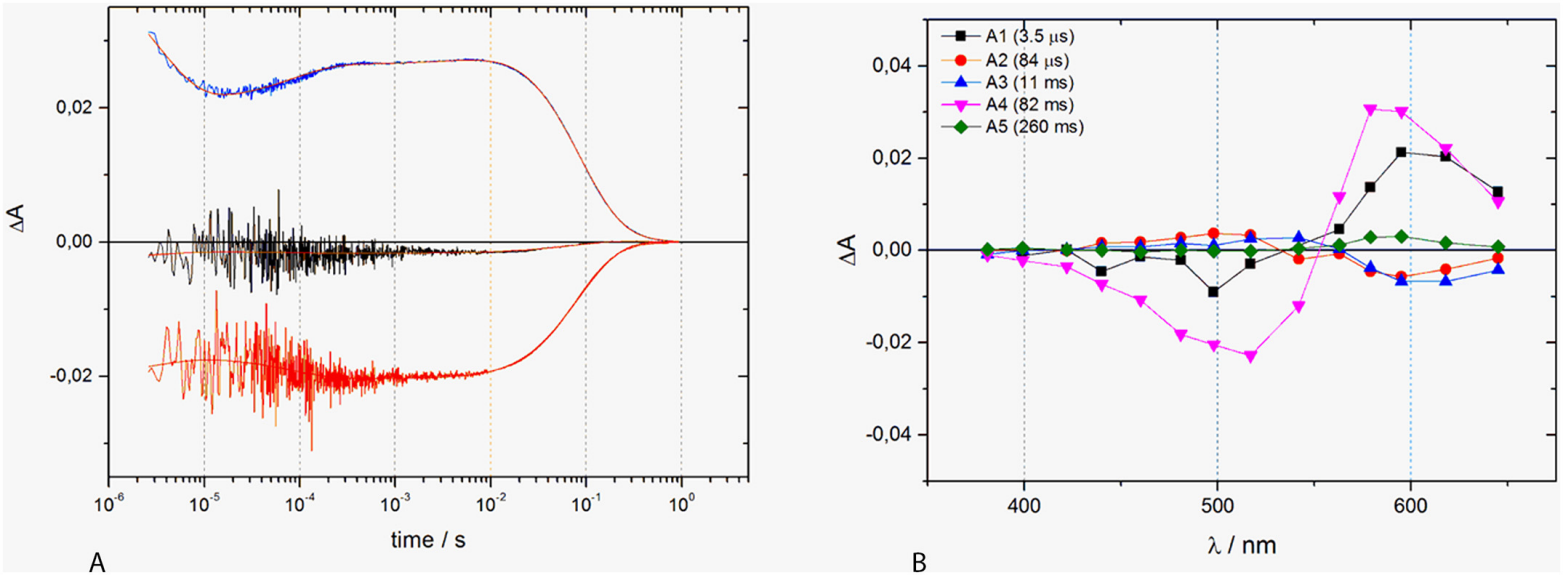
Transient absorbance changes of E17R. A. The kinetics of the absorbance changes are shown for selected wavelengths (399 nm, 517 nm and 598 nm). B. The decay-associated spectra are depicted as obtained from the global fit. The corresponding time constants are given in the figure.

Flash photolysis as a time-resolved detection method for absorption changes allows following—after a short laser pulse (routinely 10 ns)—absorbance changes at a wide wavelength range on a time domain from few microseconds into the seconds range. Detection of absorbance changes, followed by fit procedures provides details on the lifetime of intermediate states formation and decay. In most cases, the global fit approach is used where all detected absorbance changes over time and wavelengths are subjected to one single fit model. This data evaluation yields lifetimes and also spectral shape of the intermediates through which the protein travels. Here, we followed the built-up and decay of photo-intermediates from time-resolved absorbance changes in the microsecond to second time range after excitation of E17R’s initial dark state by a nanosecond laser flash (Fig 6A). The results allow us to deduce a photocycle with a lifetime of 82 ms for the recovery of the parental dark state. Global fit analysis of the kinetic traces clearly identifies formation and decay of different intermediates and the bleaching and recovery of the ground state (Fig 6B). The first intermediate is red-shifted compared to the ground state. Its formation is below the time resolution of our system. It decays partly in a fast process (τ_1_ = 3.5 μs), followed by the formation of another red-shifted intermediate. This species is built in a two-step process with small amplitudes (τ_2_ = 84 μs and τ_3_ = 11 ms). A lifetime of τ_4_ = 82 ms corresponds to the final decay of all red-shifted intermediates and the concomitant formation of the parental 524 nm-form. These flash photolysis experiments did not reveal the presence of a deprotonated species (M-state). Such behaviour is reminiscent of the ESR that also has no noticeable transient concentration of an M-state [58–60]. It was argued for the ESR protein that the absence of this intermediate is solely due to kinetics parameters, i.e., caused by similarly fast formation and decay kinetics.

### Light-induced proton transport currents

**Fig 7:**
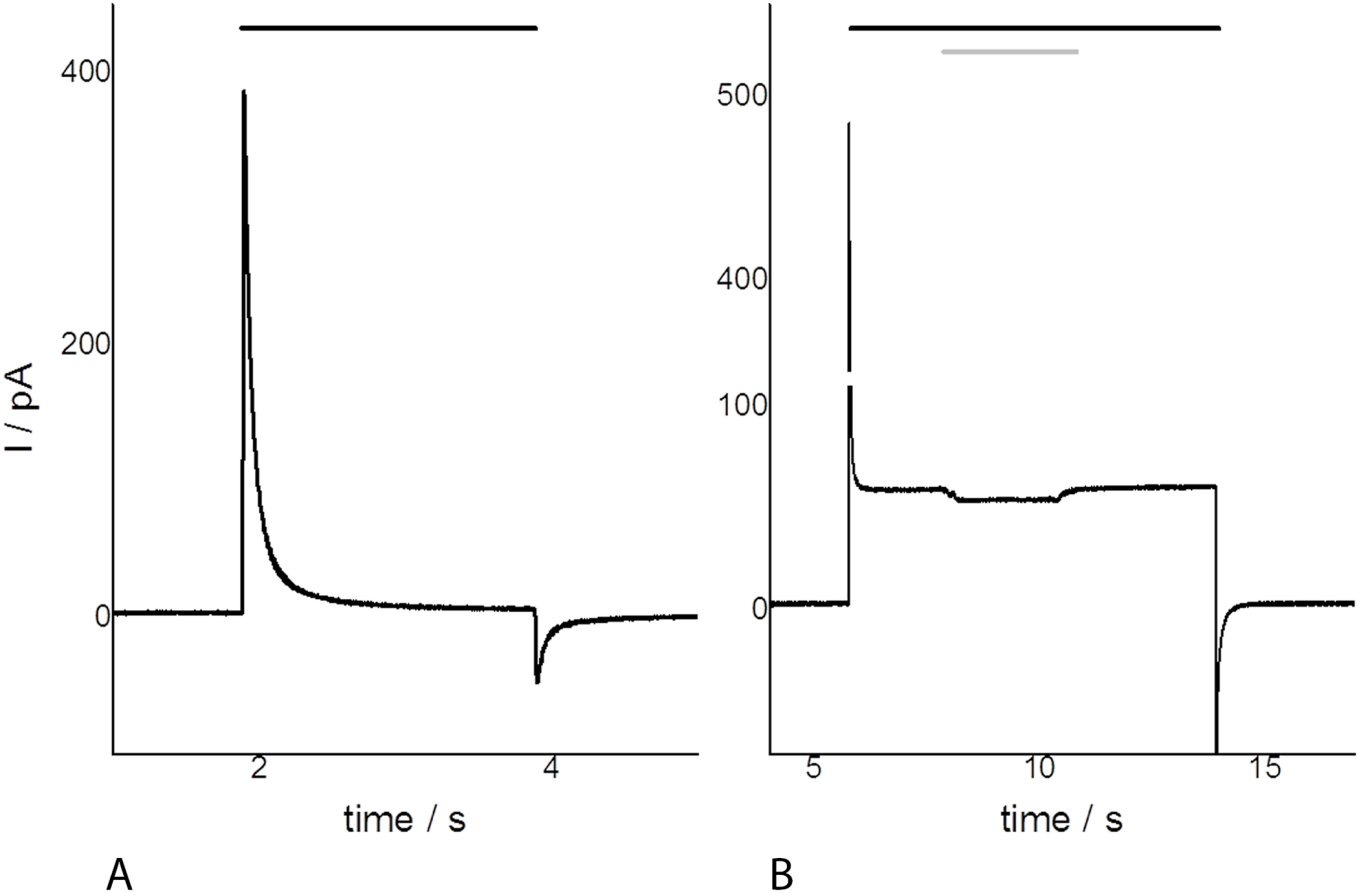
Electrometric record of E17R (in 20 mM Hepes, pH 7.4) with the BLM system. A. Transient currents and B. after the addition of the protonophore FCCP. Black bars indicate illumination with a 75 W XBO long-pass filtered at >495 nm. The grey bar shows the additional excitation of the M-state (>380 nm).

The protein pumping activity for the closely related ESR protein was measured in *E*. *coli* cell suspensions [58]. For E17R, we used a more direct proof employing the *black lipid membrane* (BLM) method [61]. E17R was reconstituted in proteoliposomes that were attached to BLM yielding capacitive coupling. Light activation generated a current of the compound membrane (Fig 7A). Doping the membrane with the protonophore FCCP makes the membrane proton selective and turns it into a DC coupled system, now also allowing measurement of stationary transport currents as a result of the light-driven proton transport (Fig 7B). The data clearly illustrates the light-driven proton translocation by E17R, but, interestingly, this method displays a blue-light effect in the photocycle after additional irradiation with blue light (Fig 6B, illumination >380 nm), and thus gives evidence for the presence of an M-like intermediate. Its presence becomes visible by the concomitant excitation of the ground state and the M-state that absorbs in the blue-/near UV region. The excitation of the M-state provides a shunt in the photocycle abolishing proton pumping which leads to a current amplitude decrease.

## Discussion

In the course of a genomic project from extremophilic microbes from HAAL, a gene (E17R) predicted as belonging to the bacteriorhodopsin family was found in the genome of *Exiguobacterium* sp. S17. This halotolerant, UV-resistant microorganism [48] was originally isolated from modern stromatolite at L. Socompa (3,570 m; Fig 1) [41] being able to grow at concentrations up to 100 mM As[V] and 5mM As[III] [45, 50]. Neighbor-Joining and maximum likelihood trees based on 16SrRNA gene sequences and MLSA of *Exiguobacterium* sp. S17 indicated that it is closely related and branched together with other mesophile strains (*Exiguobacterium* sp. 8-11-1, *Exiguobacterium pavilonensis* RW-2, *Exiguobacterium* sp. AT1b, *Exiguobacterium* sp. N139) clearly distant from psycrophilic strains clade: i.e. *Exiguobacterium sibiricum* 255–15 and *Exiguobacterium antarticum* B7 [45].

Sequence comparison and phylogenetic analysis of E17R indicated that it was a green-tuned proteorhodopsin, being the case also to all proteorhodopsins found in the so far reported *Exiguobacterium* genomes. Accordingly, the original environments of most isolates are shallow or surficial such as microbial mats, soil, rhizosphere, glacier ice, or hot springs (S1 File). Nevertheless, PRs were also present in *Exiguobacterium* strains isolated from ecological niches where light is less abundant such as marine sediments, industrial waste and even animal gastrointestinal tracts (S1 File). The ecological significance of PRs for survival under stressful conditions was evident for a *Vibrio* sp. strain in which deletion of the PR gene reduced survival during carbon starvation [64]. Light-dependent promotion of starvation survival and enhanced PR expression was also established for the marine dinoflagellate *Oxyrrhis marina* [36]. Bohorquez et al., (2012) [16] found diverse PR-like genes in microorganisms from shallow, oligotrophic hot spring waters at the Colombian high-Andes indicating that rhodopsin photosystems could be advantageous in these acidic hot springs by contributing to survival in ecosystems that receive abundant sunlight and where alternative energy sources may vary or be scarce. For the Socompa stromatolite community, the use of light to counteract stressful conditions could also be an interesting strategy especially to those communities situated at the top layer where light is fully available but where UV stress is maximal [41]. In accordance, all four available genomes isolated from stromatolite indigenous strains (from the top 5 mm) contained microbial rhodopsin-likes genes even despite belonging to distant taxonomical groups (firmicutes, proteobacteria and bacterioidetes) [18, 48, 49].

Although isolated from completely different niches (Andean stromatolites in hypersaline lakes vs. Siberian Permafrost), E17R shows highest homology to ESR for which recently a three-dimensional structure was reported [21]. Both PRs share the essential pump amino acids and show clear differences to the blue light-absorbing BPR. Common between all three proteins compared here (Fig 2) is the proton acceptor (D86, D85 in ESR), and also the close interaction with His58 (His57 in ESR, and His76 in BPR) that keeps the proton acceptor in its deprotonated state during the photocycle. Different, however, is a lysine as putative proton donor (Lys97 in E17R, Lys96 in ESR). Here, BPR resembles bacteriorhodopsin as both proteins carry an acidic amino acid as proton donor (D96 in BR, and E109 in BPR). Apparently, the protonated side chain of lysine (better coined an ammonium group) undergoes transient deprotonation and therefore functionally complements the normally present acidic side chain of, e.g., D96 in BR. Another feature shared by both green-absorbing PRs (different from BPR and BR) is an extension of the loop between helices three and four. Seven additional, nearly fully conserved amino acids- NGGFTQL (N is exchanged for A in ESR)-are present in the green absorbing PRs.

Inspection of the sequences of E17R and ESR identify positions at which amino acid are different (vertical arrows in Fig 2). In most cases aliphatic amino acids are exchanged against each other. Alternatively, polar (Thr, Ser) and acidic amino acids (Asp, Glu) were exchanged. Overall, the exchanges found in ESR are of a more polar nature compared to the corresponding positions in E17R (numbering follows E17R, exchanges in ESR are given after the dash: T76-D, G147—S, N183—K, V224—T). A more drastic change is found in ESR that carries an ion pair (K172 and D178), located at the entrance into the sixth helix at a position, where E17R carries a serine (173) and a lysine (179).

Highest conservation between all three aligned PRs is found in helix three with the proton donor and acceptor residues, except for the exchange of the acidic proton donor in BR and BPR for a lysine. In order to accomplish its function, the pK value of this lysine has to be lowered significantly compared to aqueous solution (as that of aspartate or glutamate is remarkably increased for maintaining their protonated state). Helix seven also shows a high degree of sequence similarity, except for the exchange V220 → F (E17R to ESR) and V224 → T in direct vicinity to the retinal-binding lysine.

Flash photolysis of E17R showed a photocycle comprised only of long wavelengths absorbing intermediates. From the initial photoproduct on (detection of its formation being below the time resolution of the instrument), all further intermediates show continuously increasing life times (τ_i_ = 3.5 μs, 84 μs, and 11 ms). The initial PR_524_ state is then formed with τ_4_ = 82 ms. At first glance the apparent absence of the M-intermediate in the transient absorbance changes at the measuring pH value of 7.4 is puzzling, as a de- and reprotonation is instrumental for a vectorial proton transport. In fact, the BLM measurements clearly demonstrate the presence of a photocurrent and blue light irradiation opens a short circuit pathway leading to reduce current. Thus, the lack of an M-intermediate at the pH-value chosen for this experiment is due to purely kinetic reasons (see also the pH-dependent measurements in [58]).

## Conclusions

In this work, we have successfully cloned and heterologously overexpressed a proteorhodopsin gene from a halotolerant, UV- and arsenic resistant strain originally isolated from a high-altitude Andean Lake (ca. 3,600 m). To our knowledge, this is the first report of a functional proteorhodopsin isolated from modern stromatolites. Its presence in a stromatolite and its functional, light-driven proton transport activity identifies *Exiguobacterium* sp. S17 as an important member of this complex microbial community. Further studies including knock-out strategies on E17R are in progress to link light-harvesting functionality to microbial physiology. High-throughput sequencing metagenomic projects on Socompa stromatolites are also being conducted to ultimately reveal the ecological significance of these versatile novel ion pumps in the structure and function of the microbial extremophilic communities. The studies offer a high window to study ancient phototrophic systems in homologous stromatolites of their Precambrian counterparts.

## Supporting Information

S1 File Isolation source and other relevant information on *Exiguobacterium* spp. strains in which PR –like genes were found. (DOCX)
