# Supplementary figures and images for: Functional Green-Tuned Proteorhodopsin from Modern Stromatolites
Source: PLoS One. 2016 May 17;11(5):e0154962. doi: 10.1371/journal.pone.0154962 (PMC4871484; doi:10.1371/journal.pone.0154962)

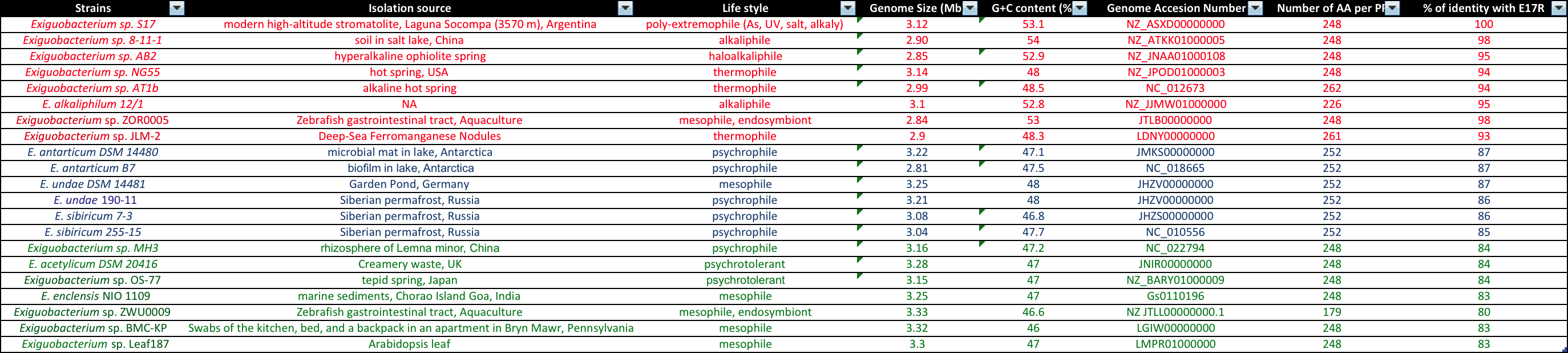

Supplement: S1 File — (DOCX) [file pone.0154962.s001.docx]
